# Self-Esteem at University: Proposal of an Artificial Neural Network Based on Resilience, Stress, and Sociodemographic Variables

**DOI:** 10.3389/fpsyg.2022.815853

**Published:** 2022-02-28

**Authors:** Juan Pedro Martínez-Ramón, Francisco Manuel Morales-Rodríguez, Cecilia Ruiz-Esteban, Inmaculada Méndez

**Affiliations:** ^1^Department of Evolutionary and Educational Psychology, Faculty of Psychology, Campus Regional Excellence Mare Nostrum, University of Murcia, Murcia, Spain; ^2^Department of Evolutionary and Educational Psychology, Faculty of Psychology, Cartuja Campus, University of Granada, Granada, Spain

**Keywords:** artificial neural network, educational psychology, professor, resilience, self-esteem, stress, university student

## Abstract

Artificial intelligence (AI) is a useful predictive tool for a wide variety of fields of knowledge. Despite this, the educational field is still an environment that lacks a variety of studies that use this type of predictive tools. In parallel, it is postulated that the levels of self-esteem in the university environment may be related to the strategies implemented to solve problems. For these reasons, the aim of this study was to analyze the levels of self-esteem presented by teaching staff and students at university (*N* = 290, 73.1% female) and to design an algorithm capable of predicting these levels on the basis of their coping strategies, resilience, and sociodemographic variables. For this purpose, the Rosenberg Self-Esteem Scale (RSES), the Perceived Stress Scale (PSS), and the Brief Resilience Scale were administered. The results showed a relevant role of resilience and stress perceived in predicting participants’ self-esteem levels. The findings highlight the usefulness of artificial neural networks for predicting psychological variables in education.

## Introduction

Self-esteem can be defined as a person’s appraisal of his or her qualities, attributions, and expectations (Ahmed et al., 2021), being of great relevance also in the academic university environment (Ferradás et al., 2020). A person with high self-esteem is a person with better socioemotional and cognitive functioning (Harun, 2017; Silverthorn et al., 2017; Kärchner et al., 2021). Low self-esteem seems to be related to academic problems and poor emotional adjustment in both the personal and social spheres; for this reason, it is necessary to measure it and take it into consideration (Kärchner et al., 2021; Zhao and Ngai, 2022). Likewise, the interaction that is generated among the members of the educational community will also affect the levels of self-esteem (Akin and Radford, 2018; Deveci and Wyatt, 2022). In line with it, self-esteem will play an important role in decision making and in the behaviors that are implemented at certain times in the university environment (Ferradás et al., 2020). Therefore, instruction in cognitive problem-solving strategies helps students improve their self-esteem (Silverthorn et al., 2017); thus, self-esteem can be modified and for this it must be previously analyzed.

In relation to resilience, self-esteem seems to play a mediating role between this concept and other psychological variables (Borji et al., 2019). Self-esteem is also a variable that has acted as a predictor of resilience in regression analyses (Balgiu, 2017). Greater resilience is associated with more functional and healthier behaviors in college students (Chacón-Cuberos et al., 2019). The emotional resources available to university professors to manage the classroom and their interaction with students should be seen as a limited resource in the sense that they can be diminished if they are overused. This point was observed when analyzing gender differences in the way of managing different situations in the classroom, finding that female teachers are more emotionally involved in interactions with students, which can also lead to greater wear and tear, while male teachers take a more formal and distant approach to relationships with a lower emotional cost that leads to saving emotional resources (Zhao and Ding, 2019). This fact alone does not explain the resilience of teachers, but it helps to understand underlying processes that may be latent and that in some way may also influence or relate to levels of self-esteem.

University can be seen as a crucial event since it involves the construction of a personal and professional life project (Herrero et al., 2019). According to these authors, who are based on well-being model of Ryff (2014), at this stage it is essential to have adequate levels of resilience to face the challenges of this educational stage. In this line, positive experiences of repeated success can contribute to a better personal valuation and to an increase in the probability of choosing resilient behaviors to reach achievement (Silverthorn et al., 2017). Among teachers, the search for meaning in teaching is one of the qualities that are most present in people with high levels of resilience (Platsidou and Daniilidou, 2021).

As can be inferred, the relationship between self-esteem and the implementation of certain behaviors to manage problems is complex (Li et al., 2018). It appears that the presence of resilient behavior and high self-esteem is related to the ability to manage emotions and self-control them (Yang et al., 2019). One mechanism that explains the generation of behavioral coping strategies in which resilience can be included is found in the sequence: thought-feeling-behavior (Silverthorn et al., 2017). In this sense, and following these authors, it seems that proactive thoughts generate pleasant feelings that are embodied at the level of observable behavior by choosing adaptive behaviors from their entire behavioral repertoire (including resilient behaviors in their case); basically, this mechanism describes a behavioral decision-making process and helps to generalize learning to natural contexts since there is a direct action of the subject with his environment. By contrast, low levels of self-esteem are associated with a greater use of avoidance behaviors that avoid confronting the problem (Silverthorn et al., 2017; Morales-Rodríguez et al., 2021), and in which it is difficult to implement resilient behavior. In addition to what has been said so far, regression analysis studies have found that self-esteem is involved in the process of predicting teachers’ perceptions of their ability to solve problems effectively (Harun, 2017).

Regarding the relationship between self-esteem and perceived stress, both concepts seem to be closely related to each other (Méndez et al., 2020). Specifically, high levels of stress may be present in situations of depressive symptomatology, anxiogenic, and even suicidal ideation, so it is important to act on all variables that are related to stress, including low self-esteem (Nguyen et al., 2019). Low levels of self-esteem have also been linked to serious consequences in teaching and university performance (Santos et al., 2017) by affecting areas related to well-being. The relationship between self-esteem and stress seems to be inverse, so that high levels of stress at work are associated with low levels of self-esteem (Nguyen et al., 2019). However, not only work factors influence the genesis of stress in teachers, but also personal variables such as resilience. The absence of resilience plays a transcendental role in the explanation of the genesis of burnout due to its predictive value for teacher stress (Ismail et al., 2020). In summary, the relationship between self-esteem and the other factors that may be affecting its levels is complex, and for this reason it is necessary to continue researching it (Ferradás et al., 2020).

Precisely to study the complex relationship between variables, artificial intelligence (AI), including artificial neural networks, is a growing technology with great implications in the field of education (Martínez-Ramón et al., 2021). In particular, AI is able to reduce human error and perform predictive analytics with less data than traditional systems, and the education system is expected to benefit from the technology surrounding this technology through more realistic decision-making processes (Colchester et al., 2016). Besides, artificial intelligence applications have a great scope for expansion, opening up new avenues for research and information analysis, exploring new paths in the field of innovation and the design of programs more in line with the educational reality (Luan et al., 2020; Dignum, 2021). Today, the specialization of AI in the educational field is leading to the concept of IAEd, which is helping teachers to simulate hypothetical scenarios and manage a large amount of data related to the teaching-learning process, school coexistence, attention to diversity, among other aspects (Schiff, 2021). This is even more critical in the wake of the pandemic resulting from COVID-19 (Martínez-Ramón et al., 2021), and the need to create online learning environments and to operate with a large amount of information (Colchester et al., 2016). Because of the novelty and multiple applications of AI and big data, it is necessary to promote studies that make use of this technology and to train the educational community in the development of applications, in the improvement of research and even, ultimately, in decision making on issues related to educational policies (Colchester et al., 2016). In addition to what has been said so far, sociodemographic variables such as age or gender can contribute to some extent to predict the cognitive processes of teachers, although their impact on artificial neural networks still requires further study (Martínez-Ramón et al., 2021; Morales-Rodríguez et al., 2021). Without a shadow of doubt, it is a problem that needs addressing.

The main objective of this research was, on the one hand, to study the relationship between resilience and stress and self-esteem and, on the other hand, to analyze the contribution of the levels of resilience and perceived stress on the levels of self-esteem of the university educational community through the design of an artificial neural network with predictive capacity. The following hypotheses were used as a starting point: (h1) Self-esteem and resilience will maintain a direct relationship so that the higher the self-esteem, the higher the resilience; (h2) self-esteem and stress will have an inversely proportional relationship so that the higher the self-esteem the lower the levels of perceived stress; and (h3) the levels of resilience and stress—at the input layer—will contribute to a greater extent to the predictive capacity of the artificial neural network of self-esteem—at the output layer.

## Materials and Methods

### Procedure

A quantitative and *ex post facto* design approach was used. Within this design, a battery of scales was administered through an online platform initially using educational communication channels to reach the target population, and once achieved, the link was allowed to continue to be shared through a snowball process, following the approach of previous studies (Morales-Rodríguez et al., 2021). The questionnaires were administered between May 2019 and May 2020. Before starting the task, the objectives of the study were informed and consent to participate was requested. The completion time was approximately 15 min. At all times the research was voluntary, confidential, and anonymous, in line with the guidelines established in the Helsinki Protocol. The study was approved by an Ethics Committee of the University of Murcia (ID: 2478/2019).

### Participants

A total of 290 people from the university educational community of two higher education centers in southeastern Spain participated in this research. Of these, 73.1% (*n* = 212) were women and 26.9% (*n* = 78) were men. Regarding their educational role at the university, 69.7% (*n* = 202) were students and 30.3% (*n* = 88) were professors. The mean age was 30.26 (*SD* = 10.784) with the mean age of females being 30.21 (*SD* = 10.746) and the mean age of males being 30.40 (*SD* = 10.954). Likewise, the mean age of the student body was 26.36 (*SD* = 8.144) and that of the faculty was 39.20 (*SD* = 10.810).

### Instruments

The instruments administered were:

*Ad hoc sociodemographic questionnaire*. For the development of this research, information was collected regarding gender (nominal variable with two options: female or male), age (continuous or scale variable), and role (nominal variable with two options: student or teacher).
*Rosenberg Self-Esteem Scale* (*RSES*). Originally designed by Rosenberg (1965) and validated in Spanish by Atienza et al. (2000), it consists of 10 items with four response options where 1 means “strongly disagree” and 4 means “strongly agree,” with direct and inverse items. Example: “In general I feel satisfied with myself” (item 7). The sum of the items gives rise to a variable that can reach 40 points. In relation to the interpretation, the higher the score, the higher the self-esteem and vice versa. Similarly, this scale was administered in university population and obtained an alpha of 0.72 (Ceballos-Ospino et al., 2017). In the current version, an *α* = 0.875 was found.
*Perceived Stress Scale* (*PSS*). The Spanish version of the stress scale (Remor, 2006) devised by Cohen et al. (1983) was administered. The scale is composed of 14 items assessed through a six-option Likert-type scale where 0 means “never” and 5 means “very frequently,” and after considering the direct and inverse items and adding them together, a total score is obtained that is directly proportional to the level of perceived stress. Example of item: “In the last month, how often have you been upset because of something that happened unexpectedly?” (Item 1). Remor (2006) found a Cronbach’s Alpha of 0.81. In the current study, 0.86 was found.
*Brief Resilient Coping Scale* (*BRCS*). The Spanish version was administered (Ceballos-Ospino et al., 2017) based in turn on the Sinclair and Wallston (2004) scale. It is rated based on a five-option scale where 1 means “It does not describe me at all” and 5 means “It describes me very well.” In total there are four items. For example, “I believe I can grow in positive ways by dealing with difficult situations” (item 3). In the original scale, a Cronbach’s Alpha of 0.86 was obtained. In the Spanish version, 0.82 was obtained. In the current research, *α* = 0.78 was obtained.

### Data Analysis

First, a descriptive analysis was carried out, analyzing the main trend and dispersion indexes. Next, an inferential analysis was performed which consisted of the application of Pearson’s correlation for continuous variables to study the relationship between variables and Student’s *t*-test to compare independent means and examine the existence of differences, and finally, an artificial neural network was designed to predict the levels of self-esteem.

Regarding the design of the artificial neural network, a backpropagation algorithm was chosen. Five independent variables consisting of two factors of a nominal nature and three covariates of a continuous nature were selected. The dependent variable was self-esteem. The scale change of covariates was standardized. When programming the artificial neural network, it was noted that the program would try to achieve a distribution of cases around 60% for the training phase, 30% for the testing phase, and 9.3% for the holdout phase. Finally, the program came close to these values and the resulting network consisted of 171 subjects in the training phase (59%), 92 subjects in the testing phase (31.7%), and 27 subjects in the holdout phase (9.3%). Then, the automatic selection of architecture was chosen according to which the minimum number of units in the hidden layer would be one and the maximum 50, taking into account the principle of simplicity and optimization. The type of network training was batch and the optimization algorithm was the scaled conjugate gradient. As for the training options, we started from an initial Lambda of 0.0000005, initial Sigma of 0.00005, and an interval shift ±0.5.

For both descriptive and inferential data analysis, the statistical package SPSS version 24 (IBM Corp., 2016) was used.

## Results

### Preliminary Analysis of the Relationship Between Self-Esteem, Perceived Stress, and Resilience

Participants obtained a mean level of self-esteem located at 31.88 (*SD* = 5.837); men obtained a mean of 31.19 (*SD* = 5.820) and women 32.14 (*SD* = 5.837) on this variable. With respect to the educational role, students obtained a mean in self-esteem of 31.80 (*SD* = 6.102) and teachers a mean of 32.07 (*SD* = 5.208).

As for perceived stress, the mean score was 26.10 (*SD* = 9.030); the mean score in men was 25.53 (*SD* = 8.644) and in women was 26.31 (*SD* = 9.179). The mean score of the students on the perceived stress variable was 26.39 (*SD* = 9.386), and that of the teachers was 25.43 (*SD* = 8.167).

In the case of resilience, they obtained a mean score of 14.993 (*SD* = 3.282); males obtained a mean score of 14.64 (*SD* = 3.369) and females of 15.03 (*SD* = 3.252). Regarding the educational role, students obtained a mean score in resilience of 14.77 (*SD* = 3.423) and teachers of 15.28 (*SD* = 2.920).

The analysis of the correlations found between self-esteem, stress, and resilience is shown in Table 1. It can be seen how there are statistically significant direct and inverse relationships between certain variables. Age was not significantly related to any of the variables in Table 1 (*p* < 0.05).

**Table 1:** Pearson correlations between self-esteem, perceived stress, and resilience (*N* = 290).

		Self-esteem	Stress	Resilience
Self-esteem	Pearson correlation	1	−0.419^**^	0.408^**^
	Sig. (two-tailed)		0.000	0.000
Stress	Pearson correlation	−0.419^**^	1	−0.400^**^
	Sig. (two-tailed)	0.000		0.000
Resilience	Pearson correlation	0.408^**^	−0.400^**^	1
	Sig. (two-tailed)	0.000	0.000	

** Correlation is significant at the 0.01 level (two-tailed).

Source: Own elaboration.

### Design of the Artificial Neural Network Architecture

**Figure 1:**
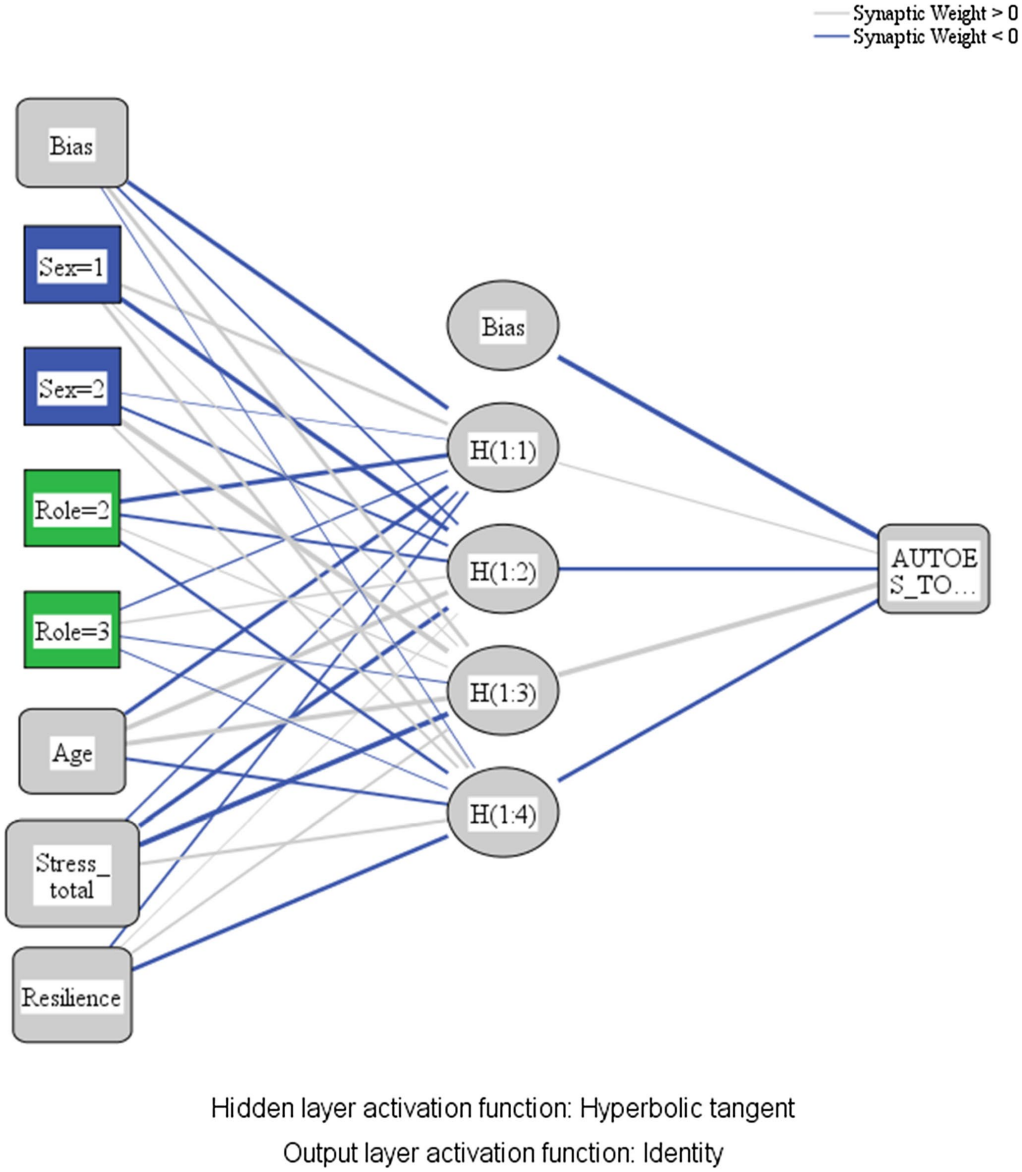
Artificial neural network. Sex = 1: Female; Sex = 2: Male; Role = 2: Student; Role = 3: Teacher; Stress_total: Total score of perceived stress; and AUTOES_TO.: Total score in self-esteem. Source: Own elaboration.

Figure 1 represents the artificial neural network model designed to predict the levels of self-esteem in the university community based on the dependent variables exposed and the calculated bias. With respect to the information in the network, it was composed of three layers (input layer, hidden layer, and output layer). First, the input layer consisted of two factors of a nominal nature (gender and educational role) and three covariates (age, perceived stress level, and resilience). The total number of units was seven excluding the bias unit and the rescaling method for covariates was standardized. Second, there is only one hidden layer consisting of four units again excluding the bias unit. Activation function was tangent hyperbolic. Third, the output layer was formed by a dependent variable (self-esteem), the rescaling method for scale dependents was standardized, the activation function was identity, and the error function was sum of squares.

In Figure 1, the lines connect the nodes and represent the relationship between them. This is called synaptic weight. Darker relationships represent values below 0, and lighter ones represent values above 0 (indirect and direct relationships respectively). The scores entered in the input layer and their interaction will determine the nodes generated in the hidden layer that will mediate the final result in the output layer. The estimated parameters of the synaptic weights of the relationships between units or nodes in the network are shown in Table 2.

**Table 2:** Estimated parameters of the artificial neural network.

Predictor		Predicted				
		Hidden layer 1				Output layer
		H(1:1)	H(1:2)	H(1:3)	H(1:4)	AUTOES_TOTAL
Input layer	(Bias)	−0.432	−0.268	0.375	−0.027	
	(Sex = 1)	0.350	−0.500	0.115	0.353	
	(Sex = 2)	−0.005	−0.300	0.789	0.288	
	(Role = 2)	−0.481	−0.302	0.115	−0.344	
	(Role = 3)	−0.150	0.203	−0.068	−0.052	
	Age	−0.403	0.537	0.562	−0.302	
	Stress_total	−0.230	−0.442	−0.650	0.327	
	Resilience	−0.270	0.017	0.282	−0.430	
Hidden layer 1	(Bias)					−0.587
	H(1:1)					0.128
	H(1:2)					−0.356
	H(1:3)					0.939
	H(1:4)					−0.426

Sex = 1: Female; Sex = 2: Male; Role = 2: Student; and Role = 3: Teacher. Source: Own elaboration.

Regarding the summary of the resulting model, the sum of squares error of the training phase was 57.177, the relative error was 0.673, and the stopping rule used was to obtain a consecutive step without decreasing the error.

In the testing phase, the sum of squares error was 32.151 and the relative error was 0.719.

In the holdout phase, the relative error was 0.943. The dependent variable was the total self-esteem score understood as a scale or quantitative variable.

The contribution of each independent variable introduced in the input layer of the artificial neural network to the final score of the dependent variable self-esteem is shown in Table 3. The importance of each variable is shown on a scale from 0 to 1 and the normalized importance from 0 to 100 (stated as a percentage). As can be seen, the sociodemographic variables (gender, educational role, and age) contribute less to the predictive capacity of the network than resilience and stress, the latter variable being the one that contributes most to explaining the subject’s self-esteem.

**Table 3:** Independent variable importance.

	Importance	Normalized importance
Sex	0.070	16.7%
Educational role	0.061	14.5%
Age	0.163	38.7%
Stress (total)	0.422	100%
Resilience (total)	0.284	67.3%

Source: Own elaboration.

**Figure 2:**
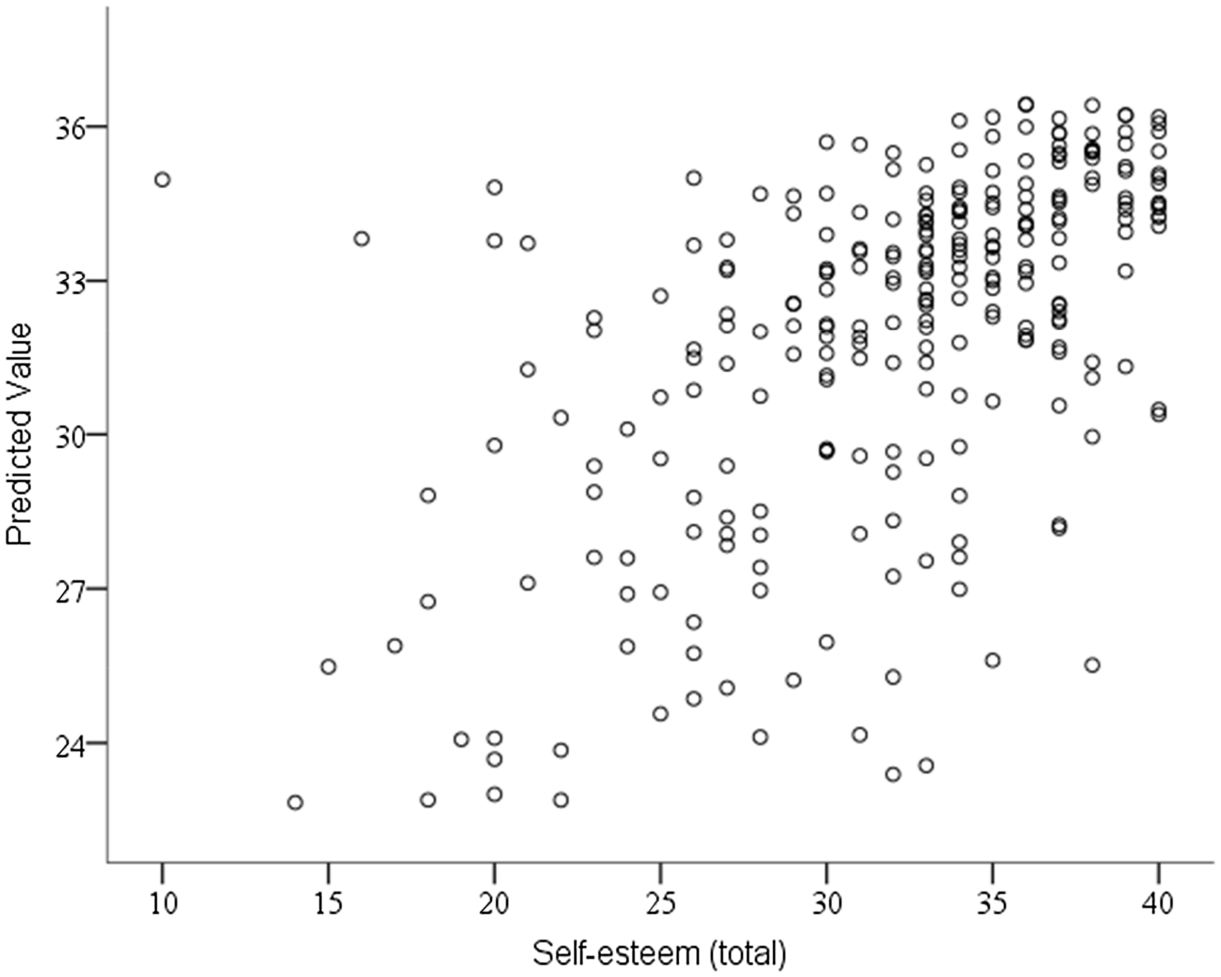
Relationship between what is predicted and what is observed by the model. Source: Own elaboration.

Figure 2 shows the comparison between what the model has predicted and what has been observed. As can be seen, an ascending cloud of points has formed in which there is a directly proportional relationship.

## Discussion

The main objective of this study was to analyze the relationship between self-esteem, stress, and resilience by designing an ANN capable of predicting the first variable as a function of the other two. With respect to hypothesis 1, it is confirmed that there is a direct relationship between self-esteem and resilience. The mechanisms that explain this relationship are varied, although it seems that resilience allows solving daily life problems and coping with high stress situations (Balgiu, 2017), which would have an impact on a higher self-esteem (Ferradás et al., 2020; Kärchner et al., 2021). In this sense, Servidio et al. (2018) affirm that those low resilient behaviors based on problem avoidance coping strategies are precisely associated with low levels of self-esteem in line also with what was raised by (Abdul et al., 2020). In short, resilient behavior has been related to higher self-esteem and adjustment (Herrero et al., 2019).

Regarding hypothesis 2, it is confirmed that there is an inverse relationship between self-esteem and stress, finding that those teachers with higher levels of self-esteem had lower levels of stress and vice versa. This is in line with what was previously stated by Santos et al. (2017), who also found that self-esteem and stress maintained an inverse relationship. Specifically, this relationship could be mediated by the coping strategies that teachers put in place (Platsidou and Daniilidou, 2021).

In relation to hypothesis 3, it is confirmed that resilience and stress have a relevant role in predicting the levels of self-esteem given an artificial neural network backpropagation algorithm, having a high contribution to its predictive capacity, above the sociodemographic variables. In the present study, stress was found to have a significance of 0.422 (normalized significance of 100%) and resilience had a significance of 0.284 (normalized significance of 67.3%). These results are closely related to those found previously in which sociodemographic variables did not provide as much predictive information to the network as the others (Martínez-Ramón et al., 2021). In any case, within the sociodemographic variables not all contribute equally. According to ANN, age contributes to self-esteem levels to a greater extent than sex or education. In any case, it was stress levels, followed by resilience, which accounted for the network results. It appears that those with lower stress levels and higher resilience have higher self-esteem. Thus, to study this relationship, artificial intelligence-derived technology has proven useful for predicting complex relationships and interactions between variables (Colchester et al., 2016; Luan et al., 2020; Martínez-Ramón et al., 2021).

The complex relationship between these variables raises the need to provide resources to increase the levels of self-esteem of the educational community as well as their strategies to cope with stress (Nguyen et al., 2019). In this scenario, teachers are a key and decisive factor in the transition from a traditional education to a digital education based on artificial intelligence, at all levels, from teaching to information management and research being their basic training to achieve educational quality and excellence (Schiff, 2021).

### Applicability and Implications

Regarding the applicability of the study, knowing which variables contribute to the levels of self-esteem of the educational community is a valuable tool to promote those factors that are more closely related to high levels of personal self-concept, which in turn are closely related to higher academic performance and lower depressive symptoms according to recent studies (Ferradás et al., 2020; Kärchner et al., 2021). It is expected that higher self-esteem favors the motivation of university students (Chacón-Cuberos et al., 2019), and this in turn leads to an increase in their academic performance (Chacón-Cuberos et al., 2021).

Likewise, the aim of this study is to provide useful information adjusted to reality in the design of intervention programs aimed at increasing self-esteem levels through a scientific and replicable approach. Such programs have been shown to have a beneficial effect on the educational community (Silverthorn et al., 2017). Such programs should focus on improving the subject’s adaptive capacity, enhancing coping strategies, and resilience (Yang et al., 2019). Because of their predictive value for teacher stress, emotional intelligence in particular, and resilience in particular, should be part of teacher training programs and their selection processes (Abdul et al., 2020).

For all these reasons, it is necessary to introduce the teaching of functional coping strategies in the curriculum itself (Silverthorn et al., 2017). At this point, educational psychology has a wide field of intervention. It is also worth mentioning knowledge transfer actions (participation in Congresses and lectures) that seem to have allowed students to feel more valued and in touch with the educational community and their teachers and raise their self-esteem levels (Akin and Radford, 2018). Therefore, these types of events should also be encouraged.

Finally, it should be noted that AI and methodology based on artificial neural networks, or more recently, AIEd, should be the basic working tool for teachers (Schiff, 2021).

### Limitations and Future Lines of Research

With regard to limitations, the network has focused on measuring self-esteem, perceived stress, and resilience, also taking into account a series of very specific sociodemographic variables, since that was the objective of this research. However, this does not mean that a person’s self-esteem at university is solely influenced by these variables. The very concept and nature of self-esteem is complex (Li et al., 2018) and further research is needed to clarify. Besides, the results should be interpreted with caution due to the number of participating subjects. In this regard, it is difficult to make generalizations to the entire university setting and ensure the representativeness of the sample, although it represents a starting point from which to continue to expand sample and encourage comparative studies across regions and countries.

With respect to future lines of study, academic performance could be included as a variable since it could also be to some extent a reflection of student self-esteem (Li et al., 2018). Likewise, further research is needed in the field of AI and in the methodologies derived from it (Luan et al., 2020). It would also be interesting to introduce coping strategies for their implication on stress and resilience levels (Platsidou and Daniilidou, 2021).

The educational field will benefit greatly from the use of AI because of the large amount of information that it must manage (Martínez-Ramón et al., 2021). There is no doubt that the main objectives of AI should be to help society to solve problems and prevent them, to promote innovation, just as these goals are also intended in the educational field. This multidisciplinary approach should involve the different sectors and social actors, including scientists, teachers, politicians, managers, etc., which requires a wide range of knowledge (i.e., financial markets, policies, education, medicine, legislation, among others; Dignum, 2021). Therefore, it will be necessary to consider what will be the new competencies of teachers in a digital world in which AI plays such an important role.

## Conclusion

Self-esteem is a complex variable. In the current research, its study has been limited to the relationship that other variables such as resilience, perceived stress, and other sociodemographic variables may have with self-esteem. Of these, stress and the individual’s resilience capacity are those that contribute most to explaining the levels of self-esteem in comparison with the sociodemographic variables. An inversely proportional relationship has also been found between perceived stress and self-esteem as well as a direct relationship between self-esteem and resilience. Gender and educational roles do not seem to have as great an influence as age when analyzing the contribution of these variables to the results in self-esteem. On the other hand, it is necessary to promote the use of methodologies based on artificial neural networks in the educational system in order to analyze the relationships between the variables that influence the teaching–learning process.

## Data Availability Statement

The raw data supporting the conclusions of this article will be made available by the authors, without undue reservation.

## Ethics Statement

The studies involving human participants were reviewed and approved by University of Murcia (ID: 2478/2019). The patients/participants provided their written informed consent to participate in this study.

## Author Contributions

All authors listed have made a substantial, direct, and intellectual contribution to the work and approved it for publication.

## Conflict of Interest

The authors declare that the research was conducted in the absence of any commercial or financial relationships that could be construed as a potential conflict of interest.

## Publisher’s Note

All claims expressed in this article are solely those of the authors and do not necessarily represent those of their affiliated organizations, or those of the publisher, the editors and the reviewers. Any product that may be evaluated in this article, or claim that may be made by its manufacturer, is not guaranteed or endorsed by the publisher.
